# Hidradenitis Suppurativa: An Interdisciplinary Problem in Dermatology, Gynecology, and Surgery—Pathogenesis, Comorbidities, and Current Treatments

**DOI:** 10.3390/life13091895

**Published:** 2023-09-11

**Authors:** Agnieszka Nowak-Liduk, Diana Kitala, Gabriela Ochała-Gierek, Wojciech Łabuś, Beata Bergler-Czop, Kornelia Pietrauszka, Paweł Niemiec, Karol Szyluk, Marcin Gierek

**Affiliations:** 1 Department of Perinatology, Gynaecology and Obstetrics, General Hospital in Ruda Śląska, Wincentego Lipa Street 2, 41-703 Ruda Śląska, Poland; agnieszkanowak3@vp.pl; 2 Dr. Stanislaw Sakiel Centre for Burns Treatment, Siemianowice Ślaskie, Jana Pawła II Street 2, 43-100 Siemianowice Śląskie, Poland; dp.kitala@gmail.com (D.K.); wojciech.labus@gmail.com (W.Ł.); 3 Department of Dermatology and Venerology, City Hospital in Sosnowiec, Zegadlowicza Street 3, 41-200 Sosnowiec, Poland; g.ochala@wp.pl; 4 Department of Dermatology, Medical University of Silesia in Katowice, Francuska Street 20-24, 40-027 Katowice, Poland; bbergler-czop@sum.edu.pl (B.B.-C.); kornelia05@wp.pl (K.P.); 5 Department of Biochemistry and Medical Genetics, School of Health Sciences in Katowice, Medical University of Silesia in Katowice, Medykow Street 18, 40-752 Katowice, Poland; pniemiec@sum.edu.pl; 6 Department of Physiotherapy, Faculty of Health Sciences in Katowice, Medical University of Silesia in Katowice, 40-752 Katowice, Poland; kszyluk@o2.pl; 7 District Hospital of Orthopaedics and Trauma Surgery, Bytomska 62 Street, 41-940 Piekary Slaskie, Poland

**Keywords:** hidradenitis suppurativa, gynecological aspects, surgical treatment, comorbidities

## Abstract

Hidradenitis suppurativa (HS), also known as acne inversa, is a chronic inflammatory disease that manifests as painful nodules, abscesses, draining dermal tunnels, and scarring in intertriginous areas such as the axillae, groin, and breasts. The nature of the disease and its chronicity have a destructive impact on mental health and quality of life. HS has an estimated global prevalence of 0.00033–4.1% and it disproportionately affects females compared to males. HS involving the female anogenital regions is reported rarely in the gynecological literature, and it can often be mistaken for other vulvar diseases. The distinct phenotypes and HS rarity cause delayed diagnosis and the implementation of effective treatment. Acne inversa is associated with several comorbidities, including metabolic disease, diabetes mellitus, inflammatory bowel diseases, and spondyloarthropathies. Although HS etiology and pathogenesis remain unclear, studies have shown that lifestyle, immunological processes, genetics, and hormonal predispositions may promote follicular hyperkeratosis, dilatation, and rupture, leading to the development of chronic tissue inflammation. This article provides updated information on HS pathogenesis, comorbidities, and treatment methods. Furthermore, we share our experience in the surgical treatment of the disease, which often proves most effective, and highlight that an interdisciplinary management approach ensures optimal outcomes.

## 1. Introduction

Hidradenitis suppurativa (HS) is characterized by recurrent painful nodules, abscesses, and draining sinus tracts, mainly in intertriginous areas [1,2]. In females, the breasts, axillae, and genito-femoral region are the most typical locations of lesions [1,2]. The inaugural International Research Symposium on HS, held in Dessau from March 30 to 2 April 2006, established the following comprehensive definition for the disease: “Hidradenitis suppurativa (HS) is a chronic, inflammatory, and recurrent skin condition primarily affecting the terminal hair follicles. It typically emerges following puberty and manifests with painful, deeply-seated, inflamed lesions in regions of the body with apocrine glands, most frequently observed in the axillary, inguinal, and anogenital areas (referred to as the Dessauer definition)” [3].

The prevalence of HS varies globally, ranging from 0.00033% to 4.1% [1]. Recent studies reported a prevalence of 0.7–1.2% in the US and European populations, though it is rare in Poland, occurring at a rate of just 0.001% [2]. In 2021, Abdulhadi et al. conducted a systematic review and meta-regression analysis involving 16 quantitatively assessed studies. Their research revealed that the overall prevalence of Hidradenitis suppurativa was determined to be 0.40% [4]. Female sufferers outnumber males at a ratio of 3:1 in Caucasian populations [1], with the disease occurring most commonly in the third and fourth decades of life. Unfortunately, the average time between disease onset and diagnosis is 7–10 years [1].

This persistent inflammatory disease has a significant impact on quality of life, causing patients to experience social stigma, poor mental health, and higher suicide rates compared to the general public [1.2]. Acne inversa is associated with a range of comorbidities, including polycystic ovary syndrome (PCOS), metabolic syndrome, diabetes mellitus type 2, mental health disorders like depression, immune-mediated diseases such as inflammatory bowel disease (IBD), spondyloarthropathies, psoriasis, alopecia areata, and thyroid dysfunction [5]. In HS patients, Diabetes mellitus (DM) stands out as perhaps the most prevalent endocrine comorbidity. Those with Hurley III classification exhibit a 5.3-fold heightened susceptibility to developing DM compared to individuals classified as Hurley I and II. As a parallel observation, it appears that addressing diabetes mellitus can also lead to an amelioration of HS symptoms, particularly in certain patients [6].

The association between Hidradenitis suppurativa (HS) and cancer is a subject of debate. Squamous cell carcinoma (SCC) is the primary type of skin cancer that has been reported in connection with HS [1]. Several HS therapeutic methods are available, including topical treatment, systemic antibiotics, hormones, biologic therapies, and surgery [5,7]. However, conservative therapy is often ineffective, and surgery is the most efficient [8]. Due to being underdiagnosed and under-treated, Hidradenitis suppurativa (HS) requires an interdisciplinary management approach to achieve optimal outcomes, considering the associated comorbidities. Consequently, HS should be regarded not only as a dermatological disorder but also as a gynecological and surgical disease [5,9].

## 2. Objectives

The primary goal of this review was to characterize HS, particularly in female patients, and highlight the gynecological aspects of the disease. As such, the paper presents current therapies in treating patients suffering from HS mainly in the anogenital area.

## 3. Materials and Methods

Between 16 February 2022, and 16 August 2023, a study was conducted, involving data collection. The researchers utilized databases such as PubMed and MedLine to search for relevant articles using specific keywords. The keywords used were (“hidradenitis suppurativa” OR “Acne inversa”) AND (“female patients” OR “woman” OR “gynaecological aspects” OR “gynecology” OR “anogenital area” OR “current treatment” OR “surgical treatment” OR “comorbidities” OR “patogenesis”).

The publication dates considered for inclusion were between 2008 and 2023. We excluded articles published before 2008 and articles written in a language other than English. The literature review encompassed original papers, case reports, and review articles written exclusively in the English language. The analysis focused on the general characteristics of the disease and the most recent treatment methods, with particular emphasis on the anogenital location of HS in women.

## 4. General Characteristics of Hidradenitis Suppurativa

To establish the diagnosis of HS, three criteria should be fulfilled to diagnose HS, including typical lesions at typical locations and chronicity. Commonly observed lesions in individuals with Hidradenitis suppurativa (HS) encompass inflammatory nodules, abscesses, sinus tracts, or fistulas that are inflamed and draining, along with distinctive rope-like scarring. The condition’s recurrence or prolonged presence can contribute to the development of additional lesion types. These encompass open comedones, bridged scars, and postinflammatory double-ended pseudocomedones, often resembling a “tombstone” in appearance. Such a range of lesions underlines the complexity and varied nature of HS manifestations [1,2,3,10]. In Hidradenitis suppurativa (HS), the array of symptoms can be categorized into primary and secondary lesions:

1. Primary lesions: follicular papule/pustule (folliculitis), nodule (inflammatory or noninflammatory), abscess
2. Secondary lesions: cyst, fistula/sinus (exudative or nonexudative), double pseudocomedone, scar (atrophic, net-like, erythematous, hypertrophic, linear, or bridged) [3].

Subcutaneous nodules and abscesses can rupture, bleed, and produce purulent discharge in a chronic process resulting in dermal contracture and skin lesion fibrosis. Anatomical locations commonly affected include the axillary, inframammary, intermammary, inguinal, perineal, perianal, and gluteal regions. The lower abdomen, suprapubic region, retroauricular areas, nape, eyelids, and scalp are less frequently reported sites. Symptoms experienced by patients may include pain, itching, malodor, a burning sensation, and local warmth [1]. Chronicity is defined as the occurrence of at least two recurrences within a six-month period [1,2]. Pruritus and discomfort often precede the development of primary lesions, particularly in areas rich in apocrine glands. The primary HS nodules can remain quiescent for days to months before evolving into abscesses that create draining sinuses with purulent discharge penetrating the skin. The healing process in HS is characterized by destructive changes that permanently alter the dermis. Sinus tracts are sealed by cordlike bands of scar tissue and fibrosis, resulting in a crisscrossing pattern of lesions and skin contractures. Interestingly, the etiology of the inflammatory cascade in HS is not bacterial in origin, as the aspirates obtained from unruptured lesions are sterile, indicating the involvement of non-infectious mechanisms in the disease process [11].

The most commonly used assessment tool for grading the severity of HS and determining the need for surgical intervention is the Hurley classification system, based on a graded scale. It is designed to help clinicians better understand disease management [11,12]:

1. Stage I: single or multiple abscess formation with no evidence of sinus tracts or scarring.
2. Stage II: recurrent abscess formation. Single or multiple abscesses are often found to be widely separated. The presence of sinus tract formation and scarring is also characteristic of the disease.
3. Stage III: affected skin typically shows diffuse or near-diffuse involvement, characterized by the presence of multiple abscesses and numerous sinus tracts throughout the affected area. These sinus tracts are often interconnected, and significant scarring is evident [11].

The initial and widely recognized Hurley Scale (1989) primarily focuses on evaluating the extent of damage inflicted by the disease rather than tracking its progression. Consequently, this scale tends to be more static in nature and might not effectively gauge the response to treatment over time. In essence, while the Hurley Scale is valuable for assessing the impact of the condition, its utility in monitoring treatment outcomes or disease evolution might be limited [12].

Over the past few years, particularly with the advancement of new targeted therapies, the count of severity scores has been consistently rising. These scores encompass a range of measures, with some being grounded in objective criteria, while others encompass a subjective assessment of factors like the patient’s pain or other grievances [12].

In 2003, Sartorius et al. introduced a novel scoring system known as the “Sartorius score” or “Original Sartorius Score”. The initial description of this score was as follows:

1. Anatomical region involved (axilla, groin, gluteal, or other region or inframammary region left and/or right: three points per region involved).
2. Number and scores of lesions (abscesses, nodules, fistulas, scars: points per lesion of all regions involved: nodules 2; fistulas 4; scars 1; others 1).
3. The longest distance between two relevant lesions, i.e., nodules and fistulas, in each region, or size if only one lesion (<5 cm, 2; <10 cm, 4; >10 cm, 8).
4. Are all lesions clearly separated by normal skin? In each region (yes 0/no 6) [12].

In 2007, Jean Revuz revisited and revised this score in an article written in French, resulting in a new version known as the “modified Sartorius Score”.

The modifications include:

1. Expanding the regions to five instead of four. This differentiation involves separating the gluteal region (convex and prone to friction) from the inter-gluteal area, which often exhibits distinct lesions.
2. Adjusting the coefficient for folliculitis-type lesions, assigning them a lower weight due to their comparatively lesser impact on daily life compared to typical inflammatory lesions.
3. Introducing the option for a “0 point” rating if there is no distance between two lesions, indicating inactive disease.

Compared to Hurley staging, the modified Sartorius Score (mSS) is notably more dynamic and capable of capturing fluctuations in severity between two consecutive visits [12].

In 2017, the European Hidradenitis Suppurativa Foundation (EHSF) members devised a user-friendly formula named the International Hidradenitis Suppurativa Severity Scoring System (IHS4). This formula involves counting the number of nodules, abscesses, and draining fistulae:

IHS4 = Nodules + 2(Abscesses) + 4(Draining fistulae)

As a result, the IHS4 score is a whole number that categorizes the disease into mild (0–3), moderate (4–10), and severe (≥11) stages [12,13,14]. This scoring system shows a strong correlation with Hurley staging, Physician Global Assessment (PGA), and the modified Sartorius Score (mSS) [12]. The novel IHS4 is a validated tool to dynamically assess HS severity and can be used both in real-life and the clinical trials setting [13,14].

To enhance the assessment of disease severity, ultrasonography was introduced. Wortsman et al. proposed a three-stage scoring system named the Sonographic Score in Hidradenitis Suppurativa (SOS-HS). Reliance solely on clinical examination can lead to an underestimation of the severity and extent of Hidradenitis suppurativa (HS). Incorporating ultrasonography (US) alongside clinical evaluation helps reveal underlying subclinical pathology and provides an anatomical insight into the scope of the lesions. Sonography enables the characterization and scoring of HS, enhancing the overall assessment of the condition [15].

Kimball et al. adopted a six-stage clinical scoring system, the Physician Global Assessment (HS-PGA), which considers the number of inflammatory nodules, abscesses, and fistulae, and is potentially applicable for sonographic evaluation [16].

The US HS-PGA aligns with anatomical definitions utilized for diagnosing and assessing HS severity in the SOS-HS and includes pseudocyst, mirroring a nodule observed during clinical examination, fluid collection, resembling an abscess, fistulous tract, akin to a fistula [16].

Nazarro et al. conducted a comparison between HS-PGA and a sonographic system, US HS-PGA, developed by themselves. They argued that US HS-PGA, in their perspective, is more accurate than SOS-HS. Their findings indicated even lower concordance between clinical and sonographic scores. The higher sensitivity of ultrasonography potentially accounts for the detection of HS lesions not evident clinically. This discordance between clinical and sonographic assessments, particularly noticeable in advanced HS stages, is likely due to the heightened sensitivity of ultrasonography [16].

Utilizing the ultrasound staging (SOS-HS) with linear probes ranging from 7 to 18 MHz for ultrasonography can significantly alter the management of Hidradenitis suppurativa (HS). Relying solely on clinical assessment may lead to an underestimation of HS severity when compared to SOS-HS evaluation. Oranges, T et al. introduced the concept of high-frequency ultrasound (HFUS) and ultra-high-frequency ultrasound (UHFUS), a promising diagnostic technique for HS. These innovative findings have the potential to enhance comprehension of the disease’s progression from early to advanced stages, ultimately enabling early diagnosis [17].

Limitations of high-frequency ultrasound (HFUS) involve the inability to detect epidermal lesions smaller than 0.1 mm and pigment. UHFUS overcomes these limitations, being able to identify lesions smaller than 0.1 mm and offering enhanced structural analysis. Although UHFUS cannot detect pigment, it offers advantages in detecting smaller lesions and analyzing their structure, a capability shared with HFUS [17].

Using ultra-high-frequency ultrasound (UHFUS) proved to be a valuable tool in enhancing our ability to identify hair follicles within fluid collections and distinguishing between pseudocysts and smaller fluid accumulations. Notably, UHFUS enabled the visualization of edema and tunnel formation even in cases where these features had not been discernible using high-frequency ultrasound (HFUS). Through UHFUS, distinct structures such as micro-tunnels, drop-shaped lesions, and microcysts became evident, marking a novel revelation in this field. Of note, the differentiation between hair tracts and micro-tunnels was more accurate with UHFUS, as HFUS tends to lack clarity in distinguishing these entities when they are smaller than 2 mm.

The identification of micro-tunnel and drop-shaped lesions possibly corresponding to hair follicles is of significance. UHFUS even managed to detect hairs within these structures, potentially signifying early pathological steps in HS. The progression from hair follicle widening to keratin plugging and the subsequent development of microcysts, potentially evolving into drop-shaped structures or pseudocysts, can be inferred from these observations [17].

This advancement in imaging methodology, especially with the newfound capability to differentiate micro-tunnels from hair tracts, holds significant potential for furthering our understanding of Hidradenitis suppurativa (HS) and its underlying pathophysiology [17].

Canoui Poitrine et al. have identified three distinct HS phenotypes that help describe the heterogeneity of the disease. These phenotypes include the axillary–mammary, follicular, and gluteal phenotypes. Each phenotype may be associated with a different etiology and prognosis, suggesting the need for distinct therapeutic approaches tailored to each phenotype [7,18]. The axillary–mammary phenotype of HS is considered the classic form described in dermatology practice. It is characterized by a high prevalence of involvement in the breast and armpit regions, as well as the presence of hypertrophic scars. On the other hand, the follicular and gluteal phenotypes are considered atypical and deviate from the more commonly observed presentation [7]. The follicular phenotype of HS primarily involves follicular lesions, including epidermal cysts, pilonidal sinuses, comedones, and severe acne. This phenotype is more commonly observed in males, smokers, and patients with more severe disease. Compared to the axillary–mammary subtype, the follicular phenotype tends to have an earlier onset and longer duration. The gluteal phenotype of HS is characterized by the presence of follicular papules and folliculitis in the gluteal region. This phenotype is more frequently observed in individuals who smoke and those with a lower body mass index (BMI). In comparison to the axillary–mammary phenotype, the gluteal phenotype tends to have decreased disease severity and a longer duration [7,18].

The outcomes of a recently conducted cross-sectional study propose that genetic alterations in the genes responsible for encoding proteins in the γ-secretase complex, notably NCSTN, may be linked to disease manifestation in a minority of Hidradenitis suppurativa (HS) patients who exhibit distinct phenotypic traits. These genetic variations seem to primarily influence patients with familial forms of HS [19].

Among the patients, some were identified as having a heterozygous condition for the NCSTN:c.671_682del variant. Notably, patients harboring this variant were more prone to experiencing prior instances of HS in unusual skin regions such as the scalp, neck, trunk, and antecubital fossa. Interestingly, even though the clinical disease severity was comparable, those with the variant exhibited a greater likelihood of needing biologic treatment. This suggests that there might be variations in treatment responses based on genetic factors [19].

## 5. Gynecological Aspects of Hidradenitis Suppurativa

In HS, there are notable gender differences in various aspects of the disease. Female patients are more likely to be obese and have first-degree relatives affected by HS. On the other hand, males often exhibit higher Hurley scores (a measure of disease severity) and higher levels of inflammatory markers. It is also observed that females tend to smoke less and generally have less severe disease compared to their male counterparts [11]. Lesions tend to affect anterior parts of the body in females (breasts, axillae, and the genito-femoral area) and often have hypertrophic scars. Meanwhile, male lesions are more common at atypical sites, such as the ears and chest, and pilonidal cysts are present. Furthermore, males with HS tend to exhibit a higher prevalence of atypical disease features, including involvement of areas such as the gluteal region, face, and back [7].

HS involving the vulva is rarely documented in the gynecological literature, and it can be easily mistaken for other vulvar diseases due to similar clinical presentations. In most cases, a clinical diagnosis can be made based on the characteristic appearance and symptoms of HS. Skin biopsies are not routinely recommended for the diagnosis of HS but may be necessary in certain situations when malignancy is suspected or when the diagnosis is uncertain. A trained obstetrician–gynecologist can diagnose HS during pelvic and breast examinations without additional skin tests [20]. Differential diagnosis in vulvar HS comprises Bartholin cysts, Crohn’s disease, and infectious diseases such as bacterial folliculitis, lymphogranuloma venerum, granuloma inguinale, and necrotizing fasciitis [20,21].

Vulvar HS presents with distinct characteristics compared to HS in other locations. It is notably associated with a higher frequency of fistulas, nodules, abscesses, the follicular phenotype, acne involving the face and thoracic region, as well as hypothyroidism. Vulvar HS is more frequent in females with a lower BMI and those with late-onset and higher incidence of psychiatric diseases. In addition, vulvar HS is negatively associated with axillary lesions [21].

According to reports, anogenital HS is associated with an increased risk of local complications resulting from disease progression. These complications may include genitourinary strictures and fistulas, limb contractures, dyspareunia, vaginal stenosis, and lymphedema in pre-existing HS [20,21]. Among the numerous HS complications, SCC development is the most severe [22]. Chronic inflammation and decreased innate immunity seen in HS have been associated with an increased risk of malignancy. Patients with HS who develop cancer typically do so after experiencing long-standing inflammation for at least 20 years [22]. Risk factors for squamous cell carcinoma (SCC) in HS include cigarette smoking and genetic predisposition. Additionally, the presence of the carcinogenic human papillomavirus (HPV) is considered a potential risk factor for the malignant transformation of HS to SCC [23,24,25]. SCC (squamous cell carcinoma) as a complication of HS tends to be more frequent in males. It predominantly develops in specific areas such as the perineal, perianal, and gluteal regions [23,24]. Indeed, early surgical excision of HS lesions is recommended to diagnose any potential occult malignant transformation [24].

In addition to the mentioned local complications, patients with hidradenitis suppurativa also experience numerous systemic complications. Patients with HS exhibit an elevated prevalence of metabolic syndrome, affecting up to 50% of cases. This connection is primarily attributed to concomitant factors such as obesity, dyslipidemia, hyperglycemia, and hypertension. Another contributing factor is the high prevalence of smoking among HS patients. This co-occurrence of metabolic syndrome and smoking potentially contributes to an augmented risk of cardiovascular-associated mortality [10,26]. Around 50% of HS patients also present with spondyloarthritis (SpA) and joint-related conditions, including osteoarthritis. The long-term consequences of chronic inflammation in HS encompass anemia, hypoproteinemia, amyloidosis, and an increased susceptibility to infectious complications like lumbosacral epidural abscess and sacral bacterial osteomyelitis [3,10,20]. Additionally, there is a heightened incidence of lymphomas, including non-Hodgkin lymphoma, Hodgkin lymphoma, and cutaneous T-cell lymphoma, among individuals with HS. These systemic effects underscore the multifaceted and potentially severe consequences associated with this condition [10].

In 2020, Al Ghamdi reported an uncommon instance of severe, chronic Hidradenitis suppurativa (HS) at Hurley stage III, localized to the vulva. This complex case featured disfiguring draining sinuses, numerous abscesses, and multiple nodules. The complications were so severe that they led to a miscarriage due to an ascending infection. The subcutaneous structure of the vulva creates a potential for rapid infection dissemination to surrounding tissues, resulting in considerable morbidity and even mortality. It has been demonstrated that ineffective antibiotic treatment or postponing essential surgical intervention for HS can have substantial negative impacts on the patient’s well-being and outcome [27].

## 6. Hidradenitis Suppurativa Pathogenesis

The etiology and pathogenesis of HS remain unclear [20,28]. Previously believed to be an inflammatory or infectious process in apocrine glands, HS is now considered a disease primarily characterized by follicular occlusion [28,29]. The exact cause of follicular plugging in HS is still a subject of debate. However, it is widely believed that immune dysregulation, genetics, hormonal fluctuations, and environmental risk factors contribute to its development [20].

Using the high-frequency ultrasound examination, the researchers demonstrated a notable occurrence of retained hair ducts, confirming the role of hair follicles as foreign bodies that induce persistent inflammation in the scalp among individuals with Hidradenitis suppurativa (HS). The ultrasound images were scrutinized to identify hair-related features, including vellus hair, which manifested as bilaminar hyperechoic linear structures that were preserved within pseudocysts, abscesses, or fistulas. These bilaminar hyperechoic segments ran parallel to the skin surface, in contrast to normal hair strands that are oriented perpendicular to the skin. Drawing from these observations and corroborated by the findings of several other published studies, we believe that there is promising evidence supporting the effectiveness of permanent hair removal utilizing a neodymium-doped Yttrium Aluminum Garnet (Nd:YAG) laser operating at a wavelength of 1064 nm. This approach has demonstrated positive outcomes in all patients with mild to moderate HS, offering hope for improved management of the condition [30].

Numerous publications confirm the significant role of the immune system in the pathogenesis of the disease. Immune dysregulation results in increased secretion of inflammatory cytokines in lesions, including tumor necrosis factor α (TNF-α), interleukins (IL), like IL-1β, IL-12, IL-23, IL-17, and antimicrobial peptides [18,24,31]. The occlusion and subsequent dilatation of the pilosebaceous unit are caused by follicular hyperkeratosis and clogging. This process, combined with the factors mentioned earlier, leads to the rupture and release of follicular contents. As a result, inflammatory reactions occur, characterized by the influx of neutrophils, lymphocytes, and histiocytes. These inflammatory responses contribute to the formation of abscesses and subsequent tissue architectural changes observed in HS [24].

In 2023, Jepsen et al. presented the outcomes of a clinical study that demonstrated the significance of the immune system in Hidradenitis suppurativa (HS). HS is recognized as an autoinflammatory disorder of keratinization, characterized by an elevated presence of B cells and plasma cells. In this study, the authors administered Fostamatinib, a spleen tyrosine kinase inhibitor targeting B cells and plasma cells, to the patients. The results indicated that Fostamatinib was well tolerated by the HS cohort, displaying no serious adverse events and contributing to improved clinical outcomes. The study highlights the potential of targeting B cells and plasma cells as a feasible therapeutic approach in HS, an avenue that merits further exploration [32].

Moreover, in a study conducted by Mintoff et al., it was discovered that serum IgG levels possess a robust discriminatory capability to differentiate Hidradenitis suppurativa (HS) patients with severe disease, as per the Hurley criteria, from cases with mild or moderate disease severity. These findings indicate the potential for serum IgG levels to serve as a biomarker of disease severity in a clinical context. Consequently, they could function as a supplementary tool to clinical severity scoring for assessing disease severity [33].

The observation that approximately one-third of HS patients have a positive family history supports the role played by genetics in disease development. One of the genetic mechanisms underlying a subgroup of familial HS is γ-secretase gene mutations [19,34,35]. The γ-secretase complex is composed of four subunits, which are intramembrane proteases. These subunits include presenilin, presenilin enhancer, nicastrin, and anterior pharynx defective 1. The genes responsible for encoding these subunits are *PSEN1/PSEN2* (presenilin 1/presenilin 2), *PSENEN* (presenilin enhancer, gamma-secretase subunit), *NCSTN* (nicastrin), *APH1A* (APH1 homolog A, gamma-secretase subunit), and *APH1B* (APH1-1 homolog B, gamma-secretase subunit). The complex is responsible for the intramembranous cleavage of various type-1 transmembrane proteins, such as amyloid precursor protein (APP) and Notch receptors. Loss-of-function mutations in the components of the γ-secretase complex can lead to the downregulation of the Notch signaling pathway. The cleavage of the Notch intracellular domain by the γ-secretase complex is essential for signal transduction through the Notch pathway. Disruption of the Notch signaling pathway has been proposed as a central factor in the pathogenesis of HS [34,36]. Indeed, dysregulation of the Notch signaling pathway has been identified not only in HS but also in other inflammatory dermatoses, including psoriasis and atopic dermatitis [37]. The genetic influences underlying HS and the associated immune dysregulation are still a topic of debate in the scientific community. However, exploring the genetic aspects of the disease may shed light on the underlying mechanisms of immune dysregulation and potentially identify targets for emerging treatments [11].

In a recently published genetic association study, it was observed that common variants associated with Hidradenitis suppurativa (HS) were situated in proximity to the SOX9 and KLF5 genes, and they were linked to the risk of developing HS. It is possible that these genes or other neighboring genes are involved in influencing the genetic susceptibility to the disease as well as the emergence of specific clinical characteristics unique to HS, like cysts, comedones, and inflammatory tunnels. The identification of these associations could offer fresh insights into the underlying mechanisms of disease pathogenesis related to these genes. This newfound understanding might enable the prediction of disease progression and could potentially lead to the development of novel treatment strategies for HS in the future [38].

## 7. Prognostic Factors in Hidradenitis Suppurativa Pathogenesis

Research in the literature supports the notion that the development of HS involves an interaction between environmental factors and genetic predisposition. The impact of obesity and tobacco smoking on the development of HS is widely recognized. Therefore, lifestyle changes, including weight management and smoking cessation, are often recommended for HS patients [34]. Within the existing literature, authors propose that serum adipokine levels experience dysregulation in the context of HS and concurrently exhibit a connection with obesity. The present study indicates a shift in adipokine expression toward the pro-inflammatory resistin and leptin [39].

In obese patients, larger skin folds can increase mechanical friction, which in turn promotes follicular occlusion and leads to the rupture of dilated follicles. Furthermore, the microenvironment within these folds, characterized by higher temperature and humidity, creates a favorable condition for bacterial proliferation. Furthermore, obesity is causally linked to a sub-acute systemic inflammatory state [29] in which adipocytes and macrophages secrete pro-inflammatory cytokines such as TNF-α, IL-1β, and IL-6 [40]. Cigarette smoking is considered a lifestyle-related factor associated with HS. Nicotine, a component of cigarettes, has been found to induce significant epidermal hyperplasia and dysbiosis, disrupt the expression of Notch-related genes, and promote biofilm formation [29].

There is no evidence to suggest that viral or fungal infections are responsible for causing HS or contribute to its pathogenesis. In fact, conventional bacterial cultures often yield negative results, or only commensal skin microbiota are identified [29]. Indeed, it has been proposed that disruption of the homeostatic symbiosis between the microbiota and the cutaneous immune system in apocrine gland-rich skin could contribute to IL-17-mediated inflammation in HS [31].

Sex hormones are believed to play a crucial role in the pathogenesis of HS, as evidenced by several observations. The higher prevalence of HS in females, the age of onset (rare before puberty and after menopause), premenstrual HS flares, changes in HS severity during pregnancy, and the efficacy of antiandrogen therapy all indicate the involvement of sex hormones in HS [34,41]. An experimental study delved into the immunohistochemical expression of the androgen receptor (AR) and estrogen receptor (ER) within HS skin tunnels. The study revealed augmented AR expression within the infundibulum and skin tunnel of HS-affected areas when contrasted with healthy skin. Notably, AR expression exhibited higher levels in males compared to females. Conversely, the immunohistochemical expression of ER was predominantly negative. Moreover, a microarray gene expression analysis demonstrated heightened androgen receptor (AR) transcriptional activity within HS lesions when compared to non-lesional skin [41].

Furthermore, sebocytes have the ability to convert dehydroepiandrosterone to testosterone within the skin itself. They also play a crucial role in regulating the expression of all three 5a-reductase isoforms, highlighting their critical involvement in skin steroidogenesis [28]. Despite the fact that most HS patients have normal androgen profiles, there have been reports of significant remission following antiandrogen therapy. It has been suggested that the pathophysiology of the disease may be correlated with enhanced peripheral androgen conversion [28].

Pregnancy generally has a beneficial effect on HS, as evidenced in the literature. Increased progesterone levels during pregnancy have been shown to inhibit the differentiation of T helper (Th) 17 cells. Furthermore, in late pregnancy, there is an increase in levels of IL-1 receptor antagonist (IL-1RA) and soluble TNF-α receptor, which neutralize the effects of TNF-α and IL-1. However, it is important to note that metabolic dysregulation and weight gain during pregnancy may worsen HS symptoms [34].

The discovery and validation of biomarkers in Hidradenitis suppurativa (HS) hold the potential to enhance the comprehension and management of this chronic and challenging condition. In a systematic review presented by Der Sarkissian et al. in 2022, a total of 48 biomarkers were identified. Among these, only four biomarkers achieved noteworthy ratings: one for diagnosis (IL-2R serum), one for monitoring (dermal Doppler vascularity), and two for prediction (epithelialized tunnels and a positive family history of HS), both of which attained a high rating [42].

However, none of the identified biomarkers displayed sufficient clinical validity to warrant their recommendation for routine use within the clinical context. The review highlighted the need for further research to address the barriers and limitations surrounding biomarkers in HS. The report concluded by outlining priorities for near-term future research aimed at enhancing the utility of biomarkers in the management of HS [42].

## 8. Comorbidities in Hidradenitis Suppurativa

HS is associated with a wide range of disorders, including type 2 diabetes mellitus, truncal obesity, polycystic ovary syndrome (PCOS), metabolic syndrome, and various diseases that exhibit a strong autoimmune component [11,39,40,43]. Notably, thyroid disease, both hyperthyroidism and hypothyroidism, have been documented in connection with HS [39].

The data indicate that nearly half of the patients demonstrated insulin resistance. Consequently, conducting insulin resistance (IR) testing in HS patients holds significance as it enables the early detection of potential diabetes mellitus. This proactive approach could play a pivotal role in averting secondary complications arising from diabetes mellitus and metabolic syndrome [39].

In the literature, the autoimmune comorbidities that are best-documented in association with HS are inflammatory bowel disease (IBD), specifically Crohn’s disease and ulcerative colitis [11].

Characterized by recurrent oral and genital ulcers, uveitis, and skin lesions, Adamantiades–Behcet disease is a chronic inflammatory vascular disorder related to HS [44]. Non-alcoholic fatty liver disease (NAFLD) is also linked to HS [7], as are pyoderma gangrenosum, seronegative spondyloarthropathies, and synovitis-acne-pustulosis-hyperostosis-osteitis (SAPHO) syndrome [43]. The latter involves arthritis and/or osteitis and chronic suppurativa skin disorders, including palmoplantar pustulosis and HS [43,45].

Other notable HS comorbidities caused by severe chronic pain and decreased quality of life include psychological problems such as anxiety, depression, and even suicidal ideation [46].

## 9. Anogenital Hidradenitis Suppurativa Treatment in Female Patients

The initial treatment of mild disease affecting the anogenital regions in female patients can be initiated by an obstetrician–gynecologist at the primary care level [20]. Recognizing and treating Hidradenitis suppurativa (HS) at an early stage can result in improved disease management and reduced morbidity for affected patients. It is crucial for gynecologists and primary care physicians to possess knowledge about vulvoperineal hidradenitis. This understanding enables them to facilitate early diagnosis, effective management, and appropriate referrals, all of which contribute to optimal outcomes for patients. Moreover, this knowledge equips gynecologists and primary care physicians to identify potential solutions for addressing vulvar deformities that might arise due to Hidradenitis suppurativa (HS) [47].

As previously mentioned, hormonal therapies, including anti-androgens and metformin, offer a therapeutic avenue for managing recalcitrant cases of Hidradenitis suppurativa (HS) or concurrent conditions linked to hormonal imbalances. This implies that it is advisable for all HS patients to undergo thorough screening by endocrinologists to identify any underlying metabolic disorders before commencing treatment. Although various associations have been observed, further research is required to shed more light on the potential hormonal dysregulation within the context of HS. A deeper understanding of these hormonal mechanisms could potentially pave the way for more effective and targeted treatment approaches for the condition [48].

However, it is strongly recommended to refer the patient suspected of HS to a dermatologist or surgeon, even at an early stage, to ensure interdisciplinary care and prevent disease progression [20]. In cases of mild to moderate HS, an obstetrician–gynecologist may prescribe various treatments. This can include topical treatment with clindamycin, oral antibiotics (such as clindamycin and rifampicin), or other adjuvant therapies like oral contraceptives, spironolactone, and metformin [20,49]. Additionally, gynecologists may perform procedures such as intralesional steroid injections (triamcinolone), excision of small localized lesions, or incision and drainage for acute pain relief, considering the high recurrence rates associated with this procedure [20,49,50].

In cases of moderate and severe HS, it is recommended to refer patients to dermatology for evaluation of immunomodulator therapy or to initiate the planning process for surgical management [20]. When conventional treatment options prove ineffective, the recommended first-line biologics for moderate-to-severe HS are monoclonal antibodies targeting TNF-α, such as adalimumab and infliximab. So far, adalimumab has been the only FDA and EMA-approved treatment for moderate-to-severe HS. An alternative option was anakinra, a recombinant IL-1RA that has shown efficacy in reducing HS severity [34,51]. Recent studies, specifically the Sunrise and Sunshine randomized trials, have examined the effectiveness of secukinumab (human monoclonal IgG1k antibody, inhibitor Interleukin 17A) in individuals with moderate-to-severe hidradenitis suppurativa. Administered biweekly, secukinumab exhibited clinical efficacy in swiftly ameliorating both the signs and symptoms of hidradenitis suppurativa [52]. Currently, secukinumab is also EMA-approved for use in patients with HS [53].

Surgical intervention should not be delayed in HS management [44]. The following is a summary in Table 1.

Local excisions, deroofing, or drainage of the abscess in the context of HS are often associated with a high risk of recurrence. These procedures typically offer only temporary relief without providing a long-term cure. Moreover, there is an increased risk of scar formation and contractures associated with such interventions [49,55]. The primary cause of high recurrence rates in HS is often attributed to inadequate debridement. Healing wounds by secondary intention can lead to unsatisfactory aesthetic outcomes, while frequent dressing changes can be burdensome for patients. Therefore, it is recommended that immediate coverage of the defect should be the standard practice, as long as there is no infection present at the time of surgery [55].

**Figure 1:**
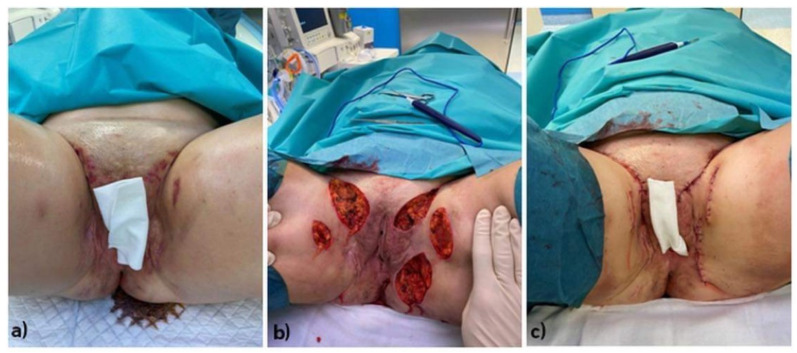
Stages of surgical treatment of a female patient with HS located on anogenital regions: (**a**) Preoperative clinical condition of the patient (pre-op), (**b**) Intraoperative clinical condition of the patient. (**c**) postoperative clinical condition of the patient. **Legend:** HS—hidradenitis syppurativa, pre-op—preoperative.

Wide local excision is considered the primary approach in traditional surgery for HS (Figure 1). This method aims to achieve a disease-free state at the excision site. However, only radical surgical excision can provide a more secure prevention of recurrence. Wound healing by secondary intention and the use of split skin grafts have been shown to be effective for this procedure [55]. In general, excision for the treatment of HS can be limited to a superficial subcutaneous plane, while deeper excision is performed based on the visible extension of the disease [49].

Reconstruction methods, including primary closure, grafts, and flaps, can expedite the healing process. However, they may be associated with higher recurrence rates compared to the natural healing process known as secondary intention healing. Grafts used in the context of reconstructive methods are typically split-thickness and employ a technique similar to that used in the treatment of significant burns. Some reports describe using ‘‘recycled skin’’ grafts or dermal scaffolds before grafting, though contour irregularities in reconstructed and donor sites are typical. Regional or free flaps can be initially bulky and may require thinning as a secondary procedure. However, they offer thicker coverage with a more natural and less scar-like appearance, which is desirable for reconstructive purposes. In ambiguous cases prior to surgical procedures, imaging techniques such as ultrasonography, magnetic resonance imaging (MRI), or fistulography play a crucial role in determining the location and depth of fistulas and sinuses [44].

Patients with HS of the groin often experience involvement of the labia, buttocks, and mons pubis. As a result, they often require customized flaps to effectively close the defects after radical excision [55]. The medial thigh-lift is a safe and reliable technique for inguinal HS that is highly accepted by patients and leads to well-hidden and mostly inconspicuous scars, good aesthetics, and a low rate of complications. The immediate wound closure and early functional recovery associated with medial thigh-lift procedures enable rapid healing and a prompt return to activity [55]. Excellent aesthetic and functional results can be achieved by combining the inferior abdominal flap and medial thigh-lift techniques for the reconstruction of large groin defects following radical excision of extensive HS [56]. Those who are not thin and have some skin laxity are considered the best candidates for utilizing the combination of the inferior abdominal flap and medial thigh-lift. To close large skin defects, reconstructive methods are often necessary [41]. Reconstructive methods in HS surgery need to be continuously improved through the use of, for example, platelet-rich plasma and acellular dermal matrix as surgical methods have their limitations [54]. Normal proregenerative mechanisms can be introduced in the human body through the use of decellularized dermal matrix (ADM). The accelular matrix created by removing cells from allogeneic human dermis is not immunogenic. It consists of extracellular matrix (ECM) structures that can be repopulated with the patient’s own cells [41]. In addition, the injection of PRP into skin flaps, along with co-grafted acellular dermal matrix and split-thickness skin grafts, has shown improvements in healing and a reduction in the number of complications [54]. Absorptive dressings are the main focus of many studies on postsurgical wound care [49]. Furthermore, negative-pressure wound therapy has demonstrated its ability to reduce the time required for excision and delayed closure or grafting [49].

## 10. Postoperative and Lifestyle Indications for Female Hidradenitis Suppurativa Patients

Comprehensive care for female patients with HS includes providing lifestyle recommendations [20]. Patients should be aware that the appropriate choice of clothing, such as garments made of 100% cotton and bamboo fiber, can help reduce mechanical friction on HS lesions [20]. Furthermore, those with vulvar, perineal, or perianal lesions should use tampons instead of sanitary napkins. Also, patients can avoid skin irritation by not shaving active flare regions. Gynecologists should consider offering an extended oral contraceptive regimen to decrease menstrual frequency. In addition, weight reduction through exercise and dietary modifications should be encouraged [20].

## 11. Summary

Skin diseases often manifest in anogenital sites, with regular reports in the literature on the gynecological aspects of skin diseases such as psoriasis, acne, and other skin changes, even during pregnancy [50,57,58]. This review aimed to provide a comprehensive update on HS epidemiology, pathogenesis, and comorbidities and highlight available treatment methods.

The issue discussed in this paper constitutes a niche, as it is an undeveloped area of research. Searching the PubMed database using keywords related to HS in gynecology returned only 38 records, which gives the impression that this serious clinical problem is underestimated. Most authors agree that HS is a debilitating disorder characterized by chronic inflammation in intertriginous areas with an often-unsatisfactory response to treatment [59]. Based on our observations, a phenomenon that worsens the prognosis further is the late diagnosis, which was confirmed by the work of Howdhury et al. [60], who pointed out the small number of cases described in the literature. Additionally, the research team of Howdhury found it necessary to establish a proper treatment plan for the disease [60]. According to other studies, it is probably even more important to attempt to understand the pathomechanisms of the disease, which would make healthcare providers and patients more aware of HS-associated risks and allow for the introduction of an appropriate treatment scheme [59,60].

It should be strongly underlined that females affected by HS should receive holistic and interdisciplinary care involving gynecologists, dermatologists, endocrinologists, radiologists, and surgeons. Moreover, it should be emphasized that only such synergistic actions result in the most desirable outcomes. Recognizing the need for a comprehensive approach to Hidradenitis suppurativa (HS) management, a group of Italian dermatologists from prominent Italian treatment centers conceived the idea of an organized framework termed the “HS Multidisciplinary Unit” (HS-MU). This unit was devised to establish a structured model incorporating various healthcare professionals capable of collaborating in the management of HS. Consensus emerged that the “Operational Core” of the HS-MU should comprise four essential healthcare experts: a dermatologist, a plastic surgeon (or a surgeon), a radiologist (or an ultrasound expert), and a nurse/wound care specialist. Additionally, diverse specialists should contribute as consultants in addressing associated comorbidities. The primary objectives of this model are to optimize the utilization of professional resources, enhance clinical outcomes, improve the quality of life for patients, facilitate patient access to HS specialists and thereby, and streamline the patient journey through the healthcare system [61].

Pharmacological treatment is limited and can only be used in Hurley Stage I, meaning there must be effective cooperation with surgeons. HS anogenital surgery is the method of choice in Hurley Stage II/III, which is confirmed in the literature [8,56,57]. Based on this literature analysis, it can be concluded that HS is often a gynecological disease, and surgical treatment is the most effective method.

By sharing our experience of treating female patients with anogenital HS, we would like to encourage clinicians to cooperate and refer patients suspected of HS to experienced specialists.

## Figures and Tables

**Table 1:** Surgical treatment in Hidradenitis suppurativa.

Technique	Method Description
Drainage	Incision of the abscess, remaining pus, rinsing with saline solution [44].
Deroofing	Removing the epithelium overlying the sinus tracts while leaving floor of the sinus and fat subcutaneous tissue [44].
STEEP	Skin-tissue-saving excision with electrosurgical peeling procedure. The affected tissue and fibrosis are peeled off and cleared from the area until it is free of lesional tissue, following an incision in the roof of the sinus [44]
Local excision	Removing tissue to the depth of subcutaneous fat to eliminate the sinus tracts [44].
Wide local excision with wound closure reconstructive techniques	- Split-thickness skin grafts. Graft of 0.6–0.8 mm thickness harvested with dermatome. Performed in one procedures after excision or previously granulated wound. - Flaps in anogenital and inguinal area: pubic lower abdominal flap (modified abdominoplasty), medial thigh-lift technique [44] - Acellular dermal matrix [41,54]
Postoperative wound care	- Negative pressure - Platelet-rich plasma

## Data Availability

The data presented in this study are available upon request from the corresponding author.

## References

[1] Nguyen T.V., Damiani G., Orenstein L.A., Hamzavi I., Jemec G. (2021). Hidradenitis suppurativa: An update on epidemiology, phenotypes, diagnosis, pathogenesis, comorbidities and quality of life. J. Eur. Acad. Dermatol. Venereol..

[2] Matusiak L., Kaszuba A., Krasowska D., Placek W., Szepietowski J. (2017). Epidemiology of hidradenitis suppurativa in Poland against the background of world data. Dermatol. Rev..

[3] Zouboulis C.C., del Marmol V., Mrowietz U., Prens E.P., Tzellos T., Jemec G.B. (2015). Hidradenitis Suppurativa/Acne Inversa: Criteria for Diagnosis, Severity Assessment, Classification and Disease Evaluation. Dermatology.

[4] Jfri A., Nassim D., O’brien E., Gulliver W., Nikolakis G., Zouboulis C.C. (2021). Prevalence of Hidradenitis Suppurativa. JAMA Dermatol..

[5] Condamina M., Penso L., Tran V.-T., Hotz C., Guillem P., Villani A.P., Perrot P., Bru M.-F., Jacquet E., Nassif A. (2021). Baseline Characteristics of a National French E-Cohort of Hidradenitis Suppurativa in ComPaRe and Comparison with Other Large Hidradenitis Suppurativa Cohorts. Dermatology.

[6] Abu Rached N., Gambichler T., Ocker L., Dietrich J., Quast D., Sieger C., Seifert C., Scheel C., Bechara F.G. (2023). Screening for Diabetes Mellitus in Patients with Hidradenitis Suppurativa—A Monocentric Study in Germany. Int. J. Mol. Sci..

[7] Canoui-Poitrine F., Le Thuaut A., Revuz J.E., Viallette C., Gabison G., Poli F., Pouget F., Wolkenstein P., Bastuji-Garin S. (2013). Identification of three hidradenitis suppurativa phenotypes: Latent class analysis of a cross-sectional study. J. Investig. Dermatol..

[8] Matusiak L., Bieniek A. (2011). Inverted acne treated surgically with innovative techniques to reduce the resulting cavities—A case report. Dermatol. Rev..

[9] Kiss N., Plázár D., Lőrincz K., Bánvőlgyi A., Valent S., Wikonkál N. (2019). Gynecological aspects of hidradenitis suppurativa. Orv. Hetil..

[10] Sabat R., Jemec G.B.E., Mayusiak Ł., Kimball A.B., Prenas E. (2020). Hidradenitis suppurativa. Nat. Rev. Dis. Primers.

[11] Preda-Naumescu A., Ahmed H.N., Mayo T.T., Yusuf N. (2021). Hidradenitis suppurativa: Pathogenesis, clinical presentation, epidemiology, and comorbid associations. Int. J. Dermatol..

[12] Daoud M., Suppa M., Benhadou F., Daxhelet M., Njimi H., White J., Jemec G., Del Marmol V. (2023). Overview and comparison of the clinical scores in hidradenitis suppurativa: A real-life clinical data. Front. Med..

[13] Zouboulis C.C., Tzellos T., Kyrgidis A., Jemec G.B.E., Bechara F.G. (2017). Development and validation of IHS4, a novel dynamic scoring system to assess hidradenitis suppurativa/acne inversa severity. Br. J. Dermatol..

[14] Zouboulis C., Matusiak L., Jemec G., Szepietowski J., Álvarez-Chinchilla P., Asoskova A., Bonnekoh H., Brattoli G., Cetinarslan T., Dawicka J. (2019). Inter-rater and intrarater agreement and reliability in clinical staging of hidradenitis suppurativa/acne inversa. Br. J. Dermatol..

[15] Wortsman X., Moreno C., Soto R., Arellano J., Pezo C., Wortsman J. (2013). Ultrasound In-Depth Characterization and Staging of Hidradenitis Suppurativa. Dermatol. Surg..

[16] Nazzaro G., Passoni E., Guanziroli E., Casazza G., Muratori S., Barbareschi M., Veraldi S., Marzano A.V. (2018). Comparison of clinical and sonographic scores in a cohort of 140 patients with hidradenitis suppurativa from an Italian referral centre: A retrospective observational study. Eur. J. Dermatol..

[17] Oranges T., Vitali S., Benincasa B., Izzetti R., Lencioni R., Caramella D., Romanelli M., Dini V. (2020). Advanced evaluation of HS with ultra-high frequency ultrasound: A promising tool for the diagnosis and monitoring of disease progression. Skin Res. Technol..

[18] Jorgensen A.H.R., Thomsen S.F., Ring H.C. (2019). Clinical phenotypes of hidradenitis suppurativa. J. Eur. Acad. Dermatol. Venereol..

[19] Mintoff D., Pace N.P., Borg I. (2023). NCSTN In-Frame Deletion in Maltese Patients With Hidradenitis Suppurativa. JAMA Dermatol..

[20] Collier E.K., Parvataneni R.K., Lowes M.A., Naik H.B., Okun M., Shi V.Y., Hsiao J.L. (2021). Diagnosis and management of hidradenitis suppurativa in women. Am. J. Obstet. Gynecol..

[21] López-Llunell C., Romaní J., Garbayo-Salmons P., Agut-Busquet E. (2021). Vulvar hidradenitis suppurativa: Clinical cross-sectional study of 25 patients. J. Dermatol..

[22] Fabbrocini G., Ruocco E., De Vita V., Monfrecola G. (2016). Squamous cell carcinoma arising in longstanding hidradenitis suppurativa: An overlooked facet of the immunocompromised district. Clin. Dermatol..

[23] Sevray M., Dupré P.-F., Le Flahec G., Trimaille A., Misery L., Brenaut E. (2019). Vulvar squamous cell carcinoma complicates hidradenitis suppurativa in a young woman. JAAD Case Rep..

[24] Vergeldt T., Driessen R., Bulten J., Nijhuis T., de Hullu J. (2022). Vulvar cancer in hidradenitis suppurativa. Gynecol. Oncol. Rep..

[25] Scheinfeld N. (2014). A case of a patient with stage III familial hidradenitis suppurativa treated with 3 courses of infliximab and died of metastatic squamous cell carcinoma. Dermatol. Online J..

[26] Cuenca-Barrales C., Montero-Vilchez T., Salvador-Rodríguez L., Sánchez-Díaz M., Arias-Santiago S., Molina-Leyva A. (2021). Implications of Hidradenitis Suppurativa Phenotypes in Cardiovascular Risk and Treatment Decisions: A Retrospective Cohort Study. Dermatology.

[27] Al Ghamdi D.S. (2020). Miscarriage as a Complication of Hidradenitis Suppurativa of the Vulva. Int. J. Womens Health.

[28] Karagiannidis I., Nikolakis G., Zouboulis C.C. (2016). Endocrinologic Aspects of Hidradenitis Suppurativa. Dermatol. Clin..

[29] Van Der Zee H.H., Laman J.D., Boer J., Prens E.J. (2012). Hidradenitis suppurativa: Viewpoint on clinical phenotyping, pathogenesis, and novel treatments. Exp. Dermatol..

[30] Nazzaro G., Zerboni R., Passoni E., Barbareschi M., Marzano A.V., Muratori S., Veraldi S. (2019). High-frequency ultrasound in hidradenitis suppurativa as rationale for permanent hair laser removal. Skin Res. Technol..

[31] Vossen A.R.J.V., van der Zee H.H., Prens E.P. (2018). Hidradenitis Suppurativa: A Systematic Review Integrating Inflammatory Pathways Into a Cohesive Pathogenic Model. Front. Immunol..

[32] Jepsen R., Edwards C., Flora A., Kozera E., Frew J.W. (2023). A proof-of-concept open-label clinical trial of spleen tyrosine kinase antagonism using fostamatinib in moderate-to-severe hidradenitis suppurativa. J. Am. Acad. Dermatol..

[33] Mintoff D., Borg I., Pace N.P. (2022). Serum Immunoglobulin G Is a Marker of Hidradenitis Suppurativa Disease Severity. Int. J. Mol. Sci..

[34] Rosi E., Fastame M.T., Scandagli I., Di Cesare A., Ricceri F., Pimpinelli N., Prignano F. (2021). Insights into the Pathogenesis of HS and Therapeutical Approaches. Biomedicines.

[35] Mintoff D., Pace N.P., Borg I. (2022). Interpreting the spectrum of gamma-secretase complex missense variation in the context of hidradenitis suppurativa—An in-silico study. Front. Genet..

[36] Li A., Peng Y., Taiclet L.M., Tanzi R.E. (2019). Analysis of hidradenitis suppurativa-linked mutations in four genes and the effects of PSEN1-P242LfsX11 on cytokine and chemokine expression in macrophages. Hum. Mol. Genet..

[37] Frew J.W., Navrazhina K. (2020). No evidence that impaired notch signaling differentiates hidradenitis suppurativa from other inflammatory skin diseases. Br. J. Dermatol..

[38] Sun Q., Broadaway K.A., Edmiston S.N., Fajgenbaum K., Miller-Fleming T., Westerkam L.L., Melendez-Gonzalez M., Bui H., Blum F.R., Levitt B. (2023). Genetic Variants Associated with Hidradenitis Suppurativa. JAMA Dermatol..

[39] Abu Rached N., Gambichler T., Dietrich J.W., Ocker L., Seifert C., Stockfleth E., Bechara F.G. (2022). The Role of Hormones in Hidradenitis Suppurativa: A Systematic Review. Int. J. Mol. Sci..

[40] Wolk K., Sabat R. (2016). Adipokines in psoriasis: An important link between skin inflammation and metabolic alterations. Rev. Endocr. Metab. Disord..

[41] Gierek M., Łabuś W., Kitala D., Lorek A., Ochała-Gierek G., Zagórska K.M., Waniczek D., Szyluk K., Niemiec P. (2022). Human Acellular Dermal Matrix in Reconstructive Surgery—A Review. Biomedicines.

[42] Der Sarkissian S., Hessam S., Kirby J.S., Lowes M.A., Mintoff D., Naik H.B., Ring H.C., Chandran N.S., Frew J.W. (2022). Identification of Biomarkers and Critical Evaluation of Biomarker Validation in Hidradenitis Suppurativa: A Systematic Review. JAMA Dermatol..

[43] Fimmel S., Zouboulis C. (2014). Comorbidities of hidradenitis suppurativa (acne inversa). Dermato-Endocrinology.

[44] Janse I., Bieniek A., Horvath B., Matusiak L. (2016). Surgical Procedures in Hidradenitis Suppurativa. Dermatol. Clin..

[45] Miller I., McAndrew R., Hamzavi I. (2016). Prevalence, risk factors, and comorbidities of hidradenitis suppurativa. Dermatol. Clin..

[46] Patel Z.S., Hoffman L.K., Buse D.C., Grinberg A.S., Afifi L., Cohen S.R., Lowes M.A., Seng E.K. (2017). Pain, psychological comorbidities, disability, and impaired qualify of life in hidradenitis suppurativa. Curr. Pain Headache Rep..

[47] Ogunleye A., Ndem I., Bui H., Sayed C. (2021). Vulvoperineal Hidradenitis Suppurativa: Diagnosis, Treatment, and Management of Deformities. Obstet. Gynecol. Surv..

[48] Karagiannidis J., Nikolakis G., Sabat R., Zouboulis C.C. (2016). Hidradenitis suppurativa/Acne inversa: An endocrine skin disorder?. Rev. Endocr. Metab. Disord..

[49] Alikhan A., Sayed C., Alavi A., Alhusayen R., Brassard A., Burkhart C., Crowell K., Eisen D.B., Gottlieb A.B., Hamzavi I. (2019). North American clinical management guidelines for hidradenitis suppurativa: A publication from the United States and Canadian Hidradenitis Suppurativa Foundations: Part I: Diagnosis, evaluation, and the use of complementary and procedural management. J. Am. Acad. Dermatol..

[50] Studzinska-Makara M., Pietrzak A., Lewicka M., Sulima M., Kowalczyk K., Michalak-Stoma A., Paszkowski T., Chodorowska G. (2013). Somatic and non-somatic problems connected with psoriasis in pregnancy. Ginekol. Pol..

[51] Zouboulis C., Bechara F., Dickinson-Blok J., Gulliver W., Horváth B., Hughes R., Kimball A., Kirby B., Martorell A., Podda M. (2019). Hidradenitis suppurativa/acne inversa: A practical framework for treatment optimization—Systematic review and recommendations from the HS ALLIANCE working group. J. Eur. Acad. Dermatol. Venereol..

[52] Kimball A.B., Jemec G.B.E., Alavi A., Reguiai Z., Gottlieb A.B., Bechara F.G., Paul C., Bourboulis E.J.G., Villani A.P., Schwinn A. (2023). Secukinumab in moderate-to-severe hidradenitis suppurativa (SUN SHINE and SUNRISE): Week 16 and week 52 results of two identical, multicentre, randomised, placebo-controlled, double-blind phase 3 trials. Lancet.

[53] European Medicines Agency. https://www.ema.europa.eu/en/medicines/human/EPAR/cosentyx.

[54] Gierek M., Klama-Baryła A., Łabuś W., Bergler-Czop B., Pietrauszka K., Niemiec P. (2023). Platelet-Rich Plasma and Acellular Dermal Matrix in the Surgical Treatment of Hidradenitis Suppurativa: A Comparative Retrospective Study. J. Clin. Med..

[55] Rieger U.M., Erba P., Pierer G., Kalbermatten F. (2009). Hidradenitis suppurativa of the groin treated by radical excision and defect closure by medial thigh lift: Aesthetic surgery meets reconstructive surgery. J. Plast. Reconstr. Aesthet. Surg..

[56] Mizukami T., Fujiwara M., Ishikawa K., Aoyama S., Fukamizu H. (2014). Reconstruction for extensive groin hidradenitis suppurativa using a combination of inferior abdominal flap and medial thigh-lift: A case report. Aesthetic Plast. Surg..

[57] Czuczwar P., Stępniak A., Goren A., Wrona W., Paszkowski T., Pawlaczyk M., Piekarska-Myślińska D., Woźniak S., Pietrzak A. (2016). Genital psoriasis: A hidden multidisciplinary problem—A review of the literature. Ginekol. Pol..

[58] Ciechanowicz P., Sikora M., Taradaj K., Ruta A., Rakowska A., Kociszewska-Najman B., Wielgoś M., Rudnicka L. (2018). Skin changes during pregnancy. Is that an important issue for pregnant women?. Ginekol. Pol..

[59] Althagafi H., Spence A.R., Czuzoj-Shulman N., Abenhaim H.A. (2022). Effect of hidradenitis suppurativa on obstetric and neonatal outcomes. J. Matern. Fetal Neonatal Med..

[60] Chowdhury T., Shankar M., Gousy N., Siddique B. (2021). A Rare Case of Job Syndrome With Autism: Complicated With Hidradenitis Suppurativa and Chronic Deep Vein Thrombosis. Cureus.

[61] Chiricozzi A., Veraldi S., Fabbrocini G., Didona B., Pasquinucci S., Micali G., Romanelli M. (2018). The Hidradenitis Suppurativa (HS) “Multidisciplinary Unit”: A rationale and practical proposal for an organised clinical approach. Eur. J. Dermatol..

